# Merger of two dispatch centres: does it improve quality and patient safety?

**DOI:** 10.1186/s13049-017-0383-z

**Published:** 2017-04-13

**Authors:** Alexandre Moser, Annette Mettler, Vincent Fuchs, Walter Hanhart, Claude-François Robert, Vincent Della Santa, Fabrice Dami

**Affiliations:** 1 Emergency Department, Hôpital Neuchâtelois, Maladière 45, 2000 Neuchâtel, Switzerland; 2 Dispatch Centre, State of Vaud (Fondation Urgences-Santé), César-Roux 31, 1005 Lausanne, Switzerland; 3 State of Neuchâtel, Public Health Office, Jacque-Louis De Pourtalès 2, 2000 Neuchâtel, Switzerland; 4 Emergency Department, Lausanne University Hospital, Lausanne, Switzerland

**Keywords:** Criteria-based dispatch centre (CBD), Over- and under-triage, Prehospital triage, Patient safety, Merger

## Abstract

**Background:**

Dispatch centres (DCs) are considered an essential but expensive component of many highly developed healthcare systems. The number of DCs in a country, region, or state is usually based on local history and often related to highly decentralised healthcare systems. Today, current technology (Global Positioning System or Internet access) abolishes the need for closeness between DCs and the population. Switzerland went from 22 DCs in 2006 to 17 today. This study describes from a quality and patient safety point of view the merger of two DCs.

**Methods:**

The study analysed the performance (over and under-triage) of two medical DCs for 12 months prior to merging and for 12 months again after the merger in 2015. Performance was measured comparing the priority level chosen by dispatcher and the severity of cases assessed by paramedics on site using the National Advisory Committee for Aeronautics (NACA) score. We ruled that NACA score > 3 (injuries/diseases which can possibly lead to deterioration of vital signs) to 7 (lethal injuries/diseases) should require a priority dispatch with lights and siren (L&S). While NACA score < 4 should require a priority dispatch without L&S. Over-triage was defined as the proportion of L&S dispatches with a NACA score < 4, and under-triage as the proportion of dispatches without L&S with a NACA > 3.

**Results:**

Prior to merging, Dispatch A had a sensitivity/specificity regarding the use of lights and sirens and severity of cases of 86%/48% with over- and under-triage rates of 78% and 5%, respectively. Dispatch B had sensitivity and specificity of 92%/20% and over- and under-triage rates of 84% and 7%, respectively. After they merged, global sensitivity/specificity reached 87%/67%, and over- and under-triage rates were 71% and 3%, respectively

**Conclusions:**

A part the potential cost advantage achieved by the merger of two DCs, it can improve the quality of services to the population, reducing over- and under-triage and the use of lights and sirens and therefore, the risk of accidents. This is especially the case when a DC with poor triage performance merges with a high-performing DC.

## Background

Dispatch centres (DCs) are considered an essential but expensive component (human resources, computer-aided dispatch systems, telecommunications hardware and software) of many highly developed healthcare systems. In Switzerland, the number of DCs is based on local history and often related to highly decentralised healthcare systems. There is a lack of evidence regarding the right population catchment size [1]. Current technology like global positioning systems (GPS) abolishes the need for closeness between DCs and the population. This is, along with the need to reduce costs and the difficulty maintaining those complex structures, one of the reasons we observe mergers and a decreasing number of European DCs [1]. Three types of dispatch systems are described in the literature: the Medical Priority Dispatch System (MPDS) [2], mainly used in Anglo-Saxon countries [3–5], the physician dispatch in France [6], and the criteria-based dispatch (CBD) used in some European countries [6, 7] as well as some North American DCs [8]. In Switzerland, only four DCs use the MPDS, while most others work with CBDs. As in other countries, tasks entrusted to Swiss DCs are not based on international consensus [9, 10] but on national rules [11].

Despite reluctance to merge DCs for non-rational or political reasons, Switzerland went from 22 DCs in 2006 to 17 in 2015 to serve a total population of 8 million inhabitants [1]. The last merger took place in January 2015 when the DC of state ‘A’ (catchment population of 768,000) took over the DC of state ‘B’ (catchment population of 178,000), resulting in a total catchment population of 946,000 inhabitants. From 2006 to 2015 the average Swiss catchment population size per dispatch evolved from 340,000 to 470,000 [1].

Priority dispatch accuracy is of prime concern in research on prehospital care and consists of optimising the match between patients’ needs and prehospital resources [12, 13], despite a missing consensus on the accepted percentage of over- and under-triage for dispatch activity. Our hypothesis is that the merger of these two DCs can have a positive impact on quality and patient safety, the most efficient dispatch offering a better services to the catchment population after the merger by reducing over and under-triage. The aim of this study was to quantify over- and under-triage before and after the merger for each state separately and for the whole catchment population (A + B) after merging.

## Method

### Setting

The Swiss healthcare system is highly decentralised, as each of the 26 states is sovereign regarding its healthcare system, including its prehospital systems (emergency medical services [EMS] and DCs).

This study was conducted throughout the states of Vaud (Dispatch A) and Neuchâtel (Dispatch B), both located in the French-speaking part of Switzerland. Dispatch A is a medical dispatch only, staffed by registered nurses and certified paramedics with at least five years of field experiences. It is a CBD system based on callers’ descriptions of symptoms [13]. Dispatchers rely on their own experience to conduct the interview. Each call is processed by the same dispatcher from the beginning (interview) to the end (dispatch) [13], and when appropriate, dispatchers deliver telephone-guided life-saving manoeuvres to bystanders [14]. Dispatch B takes care of medical, fire, and police calls and is staffed by employees without any medical background. Their task regarding medical calls consists in localising the event and then transmitting the information to one of the EMS agencies according to pre-established sectors without giving any pre-arrival instructions to the witness. As dispatcher are not trained to perform medical priority triage, to reduce risks, most interventions would run with lights and sirens (L&S). In order to simplify its system, State B proposed to merge its dispatch with State A’s, which was accepted.

In Switzerland, priority 1 (P1, immediate departure with L&S) is used in case of assumed vital risk for the patient. Priority 2 (P2, immediate departure without L&S) is used for emergencies without vital risk for the patient, and priority 3 (P3) is a delayed departure for patients requiring a transport [11, 13]. The prehospital network in both states consists of a three-tier system. Prehospital emergency physicians may be dispatched by the DC or later at the request from paramedics, either by ground or by helicopter [13].

### Study design

We retrospectively analysed the triage performance of the two DCs for 12 months prior to merging and then for 12 months after the merger. Secondary missions (inter-hospital transfers), missions aborted, and those with missing data (NACA score or priority of dispatch) were excluded.

**Fig. 1:**
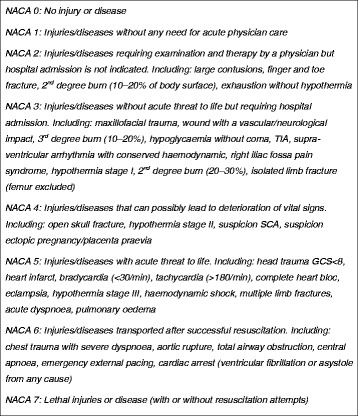
National Advisory Committee for Aeronautics (NACA) score revised by the State of Vaud (13)

The data collected from each mission were the priority decided by the dispatcher and the NACA score (National Advisory Committee for Aeronautics) (Fig 1.) [15] assessed by the prehospital crews and transmitted to the DCs at the end of the mission. The NACA scale is an eight-level scale to assess the prehospital severity status of the patient; the score is defined by the most serious clinical state experienced at any time during the mission [13] and is used in many Austrian and German EMS. In Switzerland, its use is mandatory for all prehospital missions [15]. The NACA score is significantly correlated with survival [16].

Over-triage consists of an immediate response with L&S for a non-vital condition and implies the consumption of limited resources [13], and it could also represent a danger for EMS workers and the general population while running with L&S [17], with little or no benefit to the patient [18, 19]. Under-triage is defined as an inappropriate low response without priority signs in the presence of an acute case and may be harmful for the patient [13].

According to the NACA scale (Fig. 1), a score of 4 or greater may lead to a potentially vital threat. Therefore, we assumed that those interventions are P1. All interventions with an NACA score of 1 to 3 are P2 or P3. Accordingly, P1 missions with NACA scores < 4 were classified as over-triage, and P2 and P3 missions with NACA scores >3 were classified as under-triage.

### Statistics

Simple descriptive statistics were used. Sensitivity, specificity, positive and negative predictive value (PPV & NPV), averages, percentages, and standard deviation (SD) with a 95% Confidence Interval (CI) were calculated using Microsoft Office Excel®.

### Definitions

Over-triage = P1 dispatch with NACA < 4 (false positives)/all P1 dispatch (false positives + true positives).

Under-triage = P2 or 3 dispatch with NACA > 3 (false negatives)/all P2 or P3 dispatch (false negatives + true negatives.

Sensitivity was calculated as true positives/(true positives + false negatives); specificity as true negatives/(false positives + true negatives).

Positive predictive vale (PPV) was calculated as true positives/(true positives + false positives); negative predictive value (NPV) as true negatives/(true negatives + false negatives).

## Results

Before merging, DC A totalled 27,886 primary missions (Table 1): 15,749 P1 (56.5%), 8,484 P2 (30.4%), and 3,653 P3 (13.1%). The most frequent score attributed at the end of the missions was NACA 3 (54.5%) (Table 1). Sensitivity and specificity regarding the use of L&S and severity of case were 86.0%/48.0% with over- and under-triage rates of 78.0% and 4.6%, respectively (Table 2). DC B totalled 7,791 primary missions (Table 1): 6,342 P1 (81.4%), 1,172 P2 (15.0%), and 277 P3 (3.6%). As in DC A, NACA 3 was the most frequently attributed (47.1%) (Table 1). Sensitivity and specificity were 91.6%/20.3%. Over- and under-triage rates reached 83.9% and 6.5%, respectively. Before merging there were 200 missions (0.69% of all primary dispatches) with missing data (NACA and/or priority) from DC A, and 1’139 (12.2%) from DC B.

**Table 1:** Priority dispatch and NACA score before (DC A & B) and after (A + B) merging

	Total (% of total)			P1 (% of correspondent NACA)			P2 (% of correspondent NACA)			P3 (% of correspondent NACA)		
NACA	DC A	DC B	A + B	DC A	DC B	A + B	DC A	DC B	A + B	DC A	DC B	A + B
0	322 (1.2)	334 (4.3)	617 (1.6)	184 (55.4)	245 (73.4)	206 (33.4)	128 (36.8)	69 (20.7)	370 (60.0)	20 (6.0)	20 (6.0)	41 (6.6)
1	1,247 (4.5)	462 (5.9)	2,285 (5.9)	830 (56.5)	393 (85.1)	769 (33.7)	371 (29.8)	54 (11.7)	1,326 (58.0)	46 (3.7)	15 (3.2)	190 (8.3)
2	7,122 (25.6)	2,209 (28.4)	10,639 (27.5)	4,285 (60.2)	1,899 (86.0)	3,390 (31.9)	2,228 (31.3)	261 (11.8)	5,808 (54.6)	609 (8.5)	49 (2.2)	1,441 (13.5)
3	15,208 (54.5)	3,668 (47.1)	19,995 (51.6)	7,034 (46.2)	2,781 (75.8)	6,583 (32.9)	5,290 (34.8)	704 (19.2)	9,483 (47.4)	2,884 (19.0)	183 (5.0)	3,929 (19.6)
4	2,480 (8.9)	803 (10.3)	3,490 (9.0)	2,034 (82.0)	719 (89.5)	2,888 (82.8)	376 (15.2)	76 (9.5)	517 (14.8)	70 (2.8)	8 (1.0)	85 (2.4)
5	867 (3.1)	155 (2.0)	1017 (2.6)	767 (88.5)	148 (95.5)	955 (93.9)	79 (9.1)	5 (3.2)	55 (4.4)	21 (2.4)	2 (1.3)	7 (0.7)
6	203 (0.7)	29 (0.4)	178 (0.5)	196 (96.5)	28 (96.6)	175 (98.3)	5 (2.5)	1 (3.4)	2 (1.1)	2 (1.0)	0 (0)	1 (0.6)
7	427 (1.5)	131 (1.7)	527 (1.4)	419 (98.1)	129 (98.5)	504 (95.6)	7 (1.7)	2 (1.5)	23 (4.4)	1 (0.2)	0 (0)	0 (0)
Total (% of total)	27,886 (100)	7,791 (100)	38,748 (100)	15,749 (56.5)	6,342 (81.4)	15,470 (39.9)	8,484 (30.4)	1,172 (15.0)	17,584 (45.4)	3,653 (13.1)	277 (3.6)	5,694 (14.7)

**Table 2:** Sensitivity, specificity, PPV, NPV, and under- and over-triage for DC A and B before and after merging (A + B)

	DC A % (95% CI)	DC B % (95% CI)	A + B % (95% CI)
Sensitivity	86.0 (85.6–86.4)	91.6 (91.0–92.2)	86.8 (86.5–87.1)
Specificity	48.0 (47.4–48.6)	20.3 (19.4–21.2)	67.4 (66.9–67.9)
PPV	21.7 (21.2–22.2)	16.1 (15.3–16.9)	29.2 (28.7–29.7)
NPV	95.4 (95.2–95.6)	93.5 (93.0–94.0)	97.0 (96.8–97.2)
Over-triage	78.0 (77.5–78.5)	83.9 (83.1–84.7)	70.8 (70.3–71.3)
Under-triage	4.6 (4.4–4.8)	6.5 (6.0–7.0)	3.0 (2.8–3.2)

Over-triage = P1 dispatch with NACA <4 (false positives)/all P1 dispatch (false positives + true positives) Under-triage = P2 or 3 dispatch with NACA >3 (false negatives)/all P2 or P3 dispatch (false negatives + true negatives) Sensitivity was calculated as true positives/(true positives + false negatives); specificity as true negatives/(false positives + true negatives) Positive predictive value (PPV) was calculated as true positives/(true positives + false positives); negative predictive value (NPV) as true negatives/(true negatives + false negatives)

After the merger, 38,748 missions were included (Table 1): 15,470 P1 (39.9%), 17,584 P2 (45.4%), and 5,694 P3 (14.7%). The most frequent NACA score attributed was 3 (51.6%) (Table 1). Global sensitivity/specificity reached 86.8%/67.4%, over-triage 70.8%, and under-triage 3.0% (Table 2).

Most over-triage before and after merging concern NACA 3 missions regardless of DC (Table 3). Most under-triage before and after merging concern NACA 4 missions regardless of population (Table 4). After merging, there were 5598 missions (12.2% of all primary dispatches) with missing data.

**Table 3:** Over-triage concerning NACA 3 missions before (DC A and B) and after (A + B) merging (% total over-triage)

DC A	DC B	A + B
57.0%	52.3%	60.1%

**Table 4:** Under-triage concerning NACA 4 missions before (DC A and B) and after (A + B) merging (% of total under-triage)

DC A before	DC B before	WCP after
79.5%	89.4%	87.2%

## Discussion

Following the merger, the performance of DC A showed a decrease in under-triage (3.0% vs 4.6%) without any increase in over-triage (70.8% vs 78%), a better sensitivity (86.8% vs 86.0%), and specificity (67.4% vs 48.0%) as a more restrictive use of L&S (39.9% vs 56.5%). DC A was already more efficient than DC B prior the merger but came even better after merging. The only explanation is the impact of the intensive continuous training taking place in this dispatch for many years regarding over and under-triage.

DC B’s performance compared with post-merger performance (A + B) showed a reduction in over-triage (70.8% vs 83.9%) and under-triage (3.0% vs 6.5) and improved specificity (67.4% vs 20.3%). As a result of the over-triage reduction, sensitivity decreased from 91.6% to 86.8%. The use of L&S strongly decreased from 81.4% of all missions to 39.9%.

Under-triage in dispatch may have a negative impact on patients’ safety [13]. Therefore, we can consider the reduced under-triage rate after the merger as an indicator of improved quality of services for the whole population. Over-triage is not harmful for the patients who benefit from it. It may however lead to an excessive use of L&S ambulances running hot for no or only little benefit for the patient [18, 19] and potentially fatal complications for the general population and EMS personnel [17, 20]. Over-triage may also lead to a scarcity of ambulances and endanger patients, as P1 dispatch will not be diverted to another suspected severe patient while P2 and P3 are very often in our system. We can therefore consider the reduction of the over-triage rate post-merger as an indicator not only of improved quality but also safety.

After the merger, the most frequent over-triage cases remained NACA 3 missions and under-triage cases NACA 4 missions. An explanation could be the subjectivity of the NACA score established by paramedics, and secondly, the difficulty for dispatchers, as they triage without visual cues, to differentiate future NACA 3 or 4 missions with their engagement criteria (P1, P2).

There is a lack of consensus on under- and over-triage rates in dispatch science, despite expert recommendations [21]. We should not forget that over-triage may reduce EMS capacities to respond to other patients, while eradication of under-triage is impossible without an increase of over-triage and reduced specificity.

This is due mainly to the heterogeneity of the dispatch system (CBD, MPDS, physician dispatch) and EMS system (two or three tiers), as well as to the absence of consensus on the definition of high- versus low-acuity cases [13]. The study design also plays an important role in benchmarking. Some compare dispatch priority and emergency department (ED) evaluation [22], others dispatch priority, EMS, and ED evaluations [23], and some dispatch priority and EMS evaluation [13], like in this study. As in Dami et al. [13], we decided to use the same methodology and compare only dispatch priorities and EMS field findings using the NACA score. We are deeply convinced that in a three-tier system the accuracy of priority dispatch should be evaluated by the first professional on scene for two reasons. First, it would reduce the impact of elapsed time from dispatch to clinical evaluation to its minimal, and second, an ED evaluation does not take into consideration possible improvement of the patient’s clinical condition due to EMS treatment. As stated in 2015 [13], it is still of prime importance for DCs to publish their results, as this may allow benchmarking and therefore ‘permit reaching of an international consensus on dispatch accuracy’.

This study has some limitations. It is an observational, retrospective study in a specific local setting and, as stated above, not applicable to other dispatch systems or two-tier EMS. The NACA score is known to be described as subjective [24] and not always reproducible [25], and the patient’s condition may change for better or worse while the EMS is on its way, inducing incoherence between the dispatch and EMS findings on scene. Therefore it may not always describe the patient’s clinical state on EMS arrival on site, as a minority of patient worsen during EMS care or transport, wich may overestimate under-traige. A clinical evaluation and score on EMS arrival would be the best to evaluate dispatch’s priority decision. The high rate of missing data post-merger is mainly due to omitted transmission of NACA scores. Those missing NACA scores were probably equally distributed between study groups, therefore mitigating any bias regarding over and undertriage.

## Conclusion

Apart from the potential cost advantages achieved by the merger of two DCs, it can improve the quality of services to the population, reducing over- and under-triage, increasing the availability of resources, and reducing the use of L&S and, therefore, the risk of accidents. This is especially the case when a DC with poor triage performance merges with a high-performing DC.

## Abbreviations

L&S: Lights and sirens
CBD: Criteria-based dispatch centre
CI: Confidence interval
DC: Dispatch centres
ED: Emergency department
EMS: Emergency medical services
GPS: Global Positioning System
MPDS: Medical Priority Dispatch System
NACA: National Advisory Committee for Aeronautics
NPV: Negative predictive value
P1: Priority 1
P2: Priority 2
P3: Priority 3
PPV: Positive predictive value
SD: Standard deviation
